# A Case for Consultation

**Published:** 1887-03

**Authors:** 


### A CASE FOR CONSULTATION.

S. L.

Lately I was by letters consulted in a case which offers great interest to the members of our profession.

The patient is a lawyer in the best years of manhood, hereditarily weighed down by neurasthenia. All the members of the family are of superior mental qualities, most of them not only artistically inclined, but producing works of art worthy of exhibition. I may be allowed to let the patient speak for himself:

"In the spring of 1877 I took a cold, not more serious than I had frequently experienced before. In connection with it I found myself suffering from vertigo. The cold, in a short time, did leave me, but the vertigo remained; and to my horror I discovered that I was unable to undergo any mental exertion. I had previously been indisposed to severe mental labor, but it had not reached a point where it occurred to me as being a serious matter. If now I attempted to write a simple business letter, it seemed as if the penning of each word required an exertion equal to lifting a hundred-pound weight. I had no physical pain, not even a headache, and have never suffered any since in connection with my disease. For the first four months I heard the clock strike nearly every hour in the night, and would get up feeling more tired than when I went to bed. *Muscæ volitantes* were very harassing, the shape most frequently assumed being suggestive of snakes, though, when appearing in this form, being only one at a time. The composition resembles clear drops of water joined together; also a small, dark, smoky spot, which sometimes appears separately, and sometimes in connection with the snake. The muscæ frequently appear in a bunch or spray; the dark spot not so frequent; the dark spot and clear particles always moving, not fixed. I have occasionally seen the snake-like object as far back as I can remember, but have no recollection of seeing the dark spot until now. My complexion became very florid, though I do not remember when the change took place. I consulted several physicians, but they failed to relieve me. Sometimes I found that lying on my back gave me relief, and for several years what studying I was compelled to do was done in this position. The most prominent and terrible symptom I have to contend with is suicidal mania. Sometimes one set of symptoms will predominate, and then again another. Sometimes I feel a sense of giddiness without any other noticeable symptom. Again, my mind will be very clear and yet I will suffer with the most agonizing impulse toward self-destruction. When first taken I could not keep up with a simple story told by a witness on the witness-stand. I could not carry on an ordinary social conversation, and even now it is almost impossible, when suffering from an aggravation produced from some of the medicines I have taken lately. At first I would frequently stagger, not seriously, when walking along the street, and my gait at present is often unsteady after dark. My eyes are still very sensitive to light. Two or three years after my attack I discovered sensitiveness to pressure about the third or fourth cervical vertebra. For several years I have occasionally observed that, shortly after falling asleep, I would suddenly wake with a violent jerk of the whole body. Always worse in the morning, I feel best from supper to bedtime. Not much trouble going to sleep during the forepart of the night, but wakeful during the latter part.

"One of the physicians gave me a solution of Iodine in Iodide of Potassium, which I took for several months with some benefit. He knew that there was consumption in my family, and that I, when a boy, was accustomed to expectorate considerably, and continued to do so occasionally up to the time I took the Iodine, which banished this disposition completely. Once in a great while I may catch a strong cold and cough up a little phlegm, in which case I take a dose or two of Iodine, and that is the end of it. But the effect of Iodine which pleased me most was the alleviation it gave to my nervous troubles; and in doing so it took every particle of redness out of my face. Finally, after several months, the red color returned, and I have since ascertained that about the thousandth part of a grain will produce a decided aggravation, lasting for several days.

"There are two prominent features of aggravation by different remedies: One is as if the blood in the body were collected under heavy pressure at the base of the brain, and it seems as if there were danger of explosion at that point. The mind is clear, and you know what you want to do, but something like a nightmare prevents you from doing anything speedily. The other is dizziness, difficult comprehension, and a feeling of utter imbecility and helplessness. The aggravation of Iodine causes the first.

"While traveling I had a spell of typhoid fever, a mild type, but of long duration, and while suffering from it I was very much relieved of the nervous ailment. I was up and walking around out-of-doors with my pulse ranging from ninety to one hundred beats to the minute for a month before it went down. As soon as it fell my complaint returned with renewed vigor.

"I took a high potency of Nux vom. now, as I had previously taken it low with temporary benefit, and it proved quite a relief for several months. My next experiment was with electricity. The doctor told me I had some tender spots on my body and where they were before applying the current. The spots proved to be one on each side close to the nipple, one on the breast bone just above the pit of the stomach, and another a few inches above it, one on each shoulder-blade, and one on each hip. His plan of treatment was to pass a mild current through these tender spots. I found considerable relief from its use for a while, and also noticed that it took the red color from my face, similar to Iodine, but not so effectually. I then bought a battery and went to experimenting on other members of my father's family. I found some without any tender spots, and some with a few located similarly to mine. I finally became so sensitive to the Faradic current that its slightest action would almost set me crazy, and it would turn my face to a scarlet redness. I then tried the galvanic current. Passing it mildly from the base of the brain through a portion of the spinal column gave relief; a moderate current with one pole resting on the seventh cervical vertebra and the other just under the ear takes the color out of my face and aggravates all symptoms. The Faradic current increases the redness and aggravates, the galvanic current takes out the redness and aggravates. Iodine also excites the sexual nisus, causing nocturnal emissions, and so does the Faradic current.

"While under treatment of my electrician, I discovered, on leaning back in my chair, after the aggravation became quite serious, a tender spot about the middle of the spinal column, so sensitive that it was uncomfortable to sit in a chair. I feared too much electricity had been used, discontinued it for awhile, and the tenderness passed away.

"My next trial was sea air, and I found a great deal of difference between stopping on the coast and taking a trip out to sea.

"Returning to Homœopathy, my physician tried the effect of *Phosphoric acid*, third dilution, thrice a day; about four or five doses almost made me wild. After a rest took one dose of the thirtieth, and could feel the aggravation for a week. After two or three doses of this a week apart and finding a similar excitement of the sexual powers to that produced by Iodine, I took *Thuja*[30], one dose a week. This acted finely for several weeks, but it continued to increase in power, till another change had to be made on account of the aggravation.

"*Argentum nitr.*[30], *Natr. mur.*[30], for several weeks.

"*Phosphide of zinc*[6].—An hour after taking the first dose the pavement felt as if it was filled with bumps and depressions. A week after took another dose, causing aggravation for about three weeks, rendering me very stupid.

"High potencies were now tried, but it seemed that one or two doses of any remedy upset me. My facility for observation was not so good in regard to his high potencies, and he changed now to

"*Cypripedium*[3].—One or two doses acted at first nicely, then came aggravation.

"*Senicio aur.*[3]—No better, though both exerted a moderate influence in removing the color from my face.

"*Oxygen* was recommended, and for awhile it gave promise of restoring me to health and happiness, but aggravation followed, as usual. I lost appetite, color of fæces changed to a light drab, showing very little action on the liver, though the bowels moved daily.

"Perhaps the drugs were taken too low, and I run up Cypripedium to DM, but even then it took not many doses for an aggravation. I experienced decided effects from Senecia aur[20m], but not higher.

"*Avena sativa* is the only remedy which relieved without aggravation. It acts from the tincture up to the third, but soon loses its power. Rare beef, if taken three meals in succession, aggravates and causes nocturnal emissions.

"On account of constipation, somebody praised highly hot water. I drank about three goblets, with a little sugar and milk, at each meal. In about a week I discovered that it was acting as a tonic on my nerves. A slight aggravation followed each meal, and then I would feel first rate. But the aggravation increased, and any hot food would send a thrill of electricity through my whole nervous system. It also run up my pulse to eighty or ninety, which was maintained for several weeks. My kidneys became very active, my bowels of a better color and regular, my appetite returned, and I gained in flesh. On account of a chilly sensation toward evening I consulted the doctor; he gave me a low potency of *Veratrum album*, which removed my sensitiveness to hot water and produced intense dullness and stupidity.

"I have lately run up Senecio, Cypripedium, and Phosphoric acid to the millionth. I can take Senecio and Cypripedium in the millionth for a few days without suffering great aggravations, though their effects are quite perceptible. For a number of years I have suffered with acne of the face and shoulders. A few maggot-pimples on the face, together with others containing pus. Those on the shoulders contain pus and are frequently quite large. My face also exudes in summer an undesirable quantity of oil, which is particularly noticeable upon the nose, so that it can be scraped off with a knife in considerable quantities. I have also suffered for several years with offensive perspiration of the feet.

"When a child I was very small for my age up to about thirteen, when I weighed sixty-five pounds. I then commenced growing rapidly, doubled my weight in about two years, and at sixteen weighed one hundred and fifty-two pounds. When at school I studied hard and graduated before I was seventeen years old, taking a regular classical course. Since writing the former part of my statement, I made some experiments with Chamomilla, and find that the low potencies strike me very hard, aggravating particularly the suicidal tendency. This is not noticeable to any extent when I get up into the thousands; but I dare not take more than one dose at a time, and several days apart. It also takes the color from my face to a greater extent than any other drug except Iodine.

"I do not use any tea, coffee, or chocolate, nor indulge in spices in my food. To drink chocolate two or three meals in succession produces very decided aggravation. Coffee is not so bad, but is hard upon my nerves and stomach. Black pepper produces constipation, is exciting to the sexual powers, and causes nocturnal emissions. I use very little salt with my food; acids and alkalies are very irritating to my stomach. A strong glass of lemonade will coat my tongue pretty thoroughly. A cup of strong coffee taken at a meal will coat my tongue all over heavily.

"I have been advised to eat as nourishing food as possible, especially eggs, oysters, etc. I used to eat oysters before my nervous attacks, but since then they invariably cause nausea. Fish relish only occasionally. Eggs do not agree with me very well. They suit me best cooked as hard as possible, but then they are very constipating. I can eat them raw, but fear that they produce boils. The only food to build up my nervous system which I can use to an unlimited extent is milk. This agrees with me, and I am very fond of it.

"I mentioned before of seeing a dark gray spot. Recently, on closing my eyes in the bright sunlight, I noticed what appeared to be the pupil of an eye attached to what might be considered the rest of the ball. I take this to be the gray spot that I see with my eyes open. I presume I inherit my very sensitive nervous organization from mother, who passed through some strange experience. After child-birth the room would have to be darkened so as to almost completely exclude the light. She would see sights of the strangest kind, varying from the most horrid to the most beautiful; she would hear the finest strains of music. One of the scenes that frequently occurred was a number of brilliantly burning lamps. She could always tell when she was growing better from this particular scene. The lamps grew dimmer and would then go off in smoke.

"A piece of green-grape pie will act on me as an emetic as quickly as Ipecacuanha. My general health is good, so that nobody believes in my sickness, and thus I have degenerated into a chronic misanthrope. To have struggled as hard and as diligently as I have to complete so early my course at college, and then, after studying law and being admitted to the bar, to have my health break down until I could not read a simple piece in a newspaper, and then to have people insinuate in my face that it is all mere imagination, and, what is still worse, that I was not willing to work—this puts feeling into me that devils only are supposed to carry. I presume I have said enough for all practical purposes."

—

Such was the statement which his sister brought to me with the heartfelt request to do something for a beloved brother, as accidentally I had done her so much good when given up by other physicians. After carefully reading his story, I ordered the patient to stop all medicine for six or eight weeks, follow his usual course of life, and then send in a fresh report, which here follows:

"I presume the making out of this statement may serve to illustrate one prominent symptom of my case, and that is indecision. When I have anything to do, I always feel that perhaps if I only wait awhile there may be a better opportunity or a time when I will feel in better condition to accomplish the work. This is entirely foreign to my disposition when well, for I was always very prompt and energetic, and not disposed to procrastinate.

"I find it difficult to fix my attention upon anything, am compelled to make an effort to go through the daily routine of work; feel disinclined to talk or answer questions, or to recall what I wish to, but, when suggested, I am apt to remember it; in other words, my recollection is far more at fault than my memory. At times even forget what I am about to say and have to collect my thoughts and ideas before endeavoring to express them, and while I was called a good conversationalist before my sickness, am now poor at talking at all. I am also very low spirited, and have a dread as of impending evil, though I have naturally much humor in my composition and enjoy wit. At times my perception is slow and thinking difficult; therefore lose sight of many things that go on around me—frequently in going down the street will look at persons I know well without its occurring to me until too late. Generally thinking about something else than that which is passing before me.

"I walk very fast, and generally rather impulsive in motion, and am still frequently troubled with the serpent-like bodies already mentioned. I do not like to play the woman, but at times my spirits are at such a low ebb that crying seems to be the only relief. Then again I have an irresistible desire to curse and swear, though I abhorred before even slang. Just as strongly marked is a too highly sensitive mood, and feel greatly annoyed by a too delicate consciousness.

"Nervous and restless; often wake between one and two A. M., and find sleep impossible after that time. In eating, great desire for sweets. There is a thick, tenacious mucus in my throat, rather transparent, and it interferes with my articulation. Hemorrhoids slightly, moisture about anus; want of vital heat for years, requiring at night more covering than most people, and prefer wrapping up even in day time."

—

Making a record of the symptoms, we find chiefly: Vertigo, from mental exertion; insomnia, after midnight; muscæ volitantes; suicidal mania; phthisis in ascendant; footsweats; acne pustulosa on face and neck; lying on back; morning, feels tired out; indecision of character; forgetfulness of recent events; cannot find the words to express his thoughts; inclination to weep; inclination to curse and swear; desire for sweets; want of vital heat, by covering up; loves to walk fast; piles in a slight degree.

We sent the patient a dose Anacardium orient. high. Advice is respectfully requested.

